# Gait analysis does not correlate with clinical and MR imaging parameters in patients with symptomatic lumbar spinal stenosis

**DOI:** 10.1186/1471-2474-9-89

**Published:** 2008-06-20

**Authors:** Felix Zeifang, Marcus Schiltenwolf, Rainer Abel, Babak Moradi

**Affiliations:** 1 Department of Orthopaedic Surgery, University Clinic Heidelberg, Germany; 2 Department of Orthopaedic Surgery, Hohe Warte Clinic Bayreuth, Germany

## Abstract

**Background:**

Parameters of MR imaging play a pivotal role in diagnosing lumbar spinal stenosis (LSS), and serve as an important tool in clinical decision-making. Despite the importance of MR imaging, little is known about the correlation between MRI parameters, objective gait analysis, and clinical presentation of patients with lumbar spinal stenosis.

**Methods:**

Sixty-three patients from our clinic with symptomatic lumbar spinal stenosis leading to neurogenic claudication were included in this study in accordance with clearly defined inclusion and exclusion criteria. Clinical parameters, the depression status (CES-D), the subjective functional back capacity (FFbH-R), and the absolute walking distance (treadmill gait analysis) were quantitatively evaluated in correlation with morphological data from radiographs and MRI scans, in order to determine the coherence of spinal canal narrowing and clinical affliction.

**Results:**

Sixty-three consecutive paents with a median age of 68 years and a mean Body Mass Index (BMI) of 28 were included in the study. The mean FFbH-R score displayed a value of 44 percent. The depression status scored an average of 13.6. Objectively measured walking distances showed a mean value of 172 m until patients stopped due to leg pain. A significant difference was found between the objectively measured and the subjectively estimated walking distance. The mean cross-sectional area of the dural tube at L1/2 was 113 mm^2^, at L2/3 94 mm^2^, at L3/4 73 mm^2^, at L4/5 65 mm^2^, and at L5/S1 93 mm^2^. The mean overall cross sectional area of the dural tube of all segments did not correlate with the objectively measured walking distance. However, bivariate analysis found that the BMI (tau b = -0.194), functional back capacity (tau b = -0.225), and the cross sectional area of the dural tube at L1/2 (tau b = -0.188) correlated significantly with the objectively measured walking distance.

**Conclusion:**

According to the results of this study MRI findings failed to show a major clinical relevance when evaluating the walking distance in patients with lumbar spinal stenosis and, therefore, should be treated with some caution as a predictor of walking distance. In determining the disease pattern of spinal stenosis functional back capacity and BMI might play a more active role than previously thought.

## Background

Narrowing of the spinal canal, referred to as lumbar spinal stenosis, is a rising phenomenon due to aging of the population, and has been diagnosed increasingly in the last two decades. The pathology of this disease is most typically due to degenerative changes [1-6]. Studies evaluating the canal diameter of the dural sac demonstrated that patients with spinal canal narrowing can also remain asymptomatic, concluding that the narrowing on its own should be viewed as a radiological finding without implying symptoms or prognosis. In symptomatic cases it is a painful and disabling disease most frequently affecting the elderly population. Due to the aging of the population it has become the most frequent indication for spinal surgery in patients older than 65 years.

Lumbar spinal stenosis is a common source of back and lower extremity pain accompanied by further neurological symptoms. The most predominant symptom is a history of limited walking distance, referred to as neurogenic intermittent claudication, which is generally described as pain in the lower extremities, aggravated by walking and lumbar extension and alleviated with lumbar flexion.

Despite the increasing socioeconomic impact of lumbar spinal stenosis, with its associated disabilities and costs, it remains difficult to make an accurate diagnosis. In the process of clinical decision-making, physicians rely on physical examination, the history of neurogenic claudication and imaging studies to formulate clinical diagnosis and decide further therapeutic treatment.

Even though diagnostic imaging (radiographs and MRI scans) continues to play a pivotal role in the diagnosis and clinical decision making, correlations between clinical symptoms and morphological findings are often nonspecific and, up to now cannot be clearly demonstrated [1,5,7,8]. Moreover, factors like obesity and depression seem to be associated with a worse functional status [7,9,10].

The objective of this study was to analyze the correlation between the objectively measured walking distance and the cross sectional area of the dural tube, assessed by MR imaging in patients with symptomatic lumbar spinal stenosis. In addition, the influence of clinical and sociodemographic parameters like body mass index, age, depression, and functional capacity on the walking capacity were assessed and evaluated in the context of therapeutic approaches.

## Methods

At the beginning of January 2001, 63 consecutive patients were recruited into a prospective clinical trial. The inclusion criteria were: symptoms of leg pain and aggravation due to walking, incurred over a period greater than six month, in addition to MR imaging displaying signs of stenosis of the spinal canal. A clinically relevant peripheral stenosis, as opposed to a central spinal stenosis, could be excluded in all patients on the basis of leg pain rather than radicular symptoms.

The exclusion criteria were: clinically manifest peripheral circulatory disorders assessed by sonography, which was performed on all patients except those who had already undergone angiography; joint arthritis in the lower extremities, especially hips and knees as assessed by physical examination and, if required, radiographs; polyneuropathy based on the physical examination and, if required, EMG analysis; degenerative scoliosis determined by radiographs according to Cobb with curvation above 10°; degenerative spondylolisthesis greater than 5 mm determined by radiographs; previously performed spinal surgery.

Patients subjective functional capacity were assessed by the estimation of the maximum walking distance (meters) and the Hannover Back Pain Activity Score (FFbH-R), that measures pain-related disability in musculoskeletal disorders associated with back pain prior to gait analysis [11]. This validated questionnaire is based on the Roland-Morris Questionnaire and consists of 12 items (e.g. to sit on a chair for more than 1 h), representing physical activities of daily life [12]. Greater levels of disability are reflected by higher numbers on a 24-point scale, and the overall score is expressed as an aggregate value of functional capacity. The resulting FFbH-R scores can range between 0 (no functional capacity) and 100 (maximal functional capacity) percent. The level of depressive disorders was evaluated using the German equivalent of the Center for Epidemiological Studies Depression Scale (CES-D) known as Allgemeine Depressionsskala (ADS). With this screening instrument the depression status can be assessed using a four point Likert scale; a score of 16 or higher reflects prevalent depressive symptoms.

Quantitative evaluation of locomotion was assessed with treadmill gait analysis (Treadmill Loko S70, Fa. Woodway, Weil am Rhein, Germany), conducted by physicians or physiotherapists not involved in the patients' medical treatment. The treadmill speed was adjusted to operate at each individual patients' walking speed. Standardized phrases for speaking to the patient were used, and patients were encouraged to reach their maximum walking distance. Walking distance (m), real-time (sec), and maximum walking speed (m/s) were assessed. According to the study protocol, evaluation was performed under supervision and terminated after 20 minutes, or on the patient's request if pain, fatigue, palsy, or cardiovascular complaints were observed.

Morphological data for all patients was obtained from diagnostic imaging (MRI scans). All MRI scans were evaluated by two independent clinical examiners (first and senior author) whom were unaware of each others results and those obtained from gait analysis. The overall interobserver agreement documents reasonable reliability (kappa 0.62).

The MRI scans included sagittal and axial T2-weighted images (repetition time = 3000, echo time = 98–102) from the first lumbar through the first sacral level. Scan thickness was either 4 or 5 mm, and the field of view varied from 10–15 mm. Magnetic resonance imaging based on cross-sectional images of the dural tube were obtained in accordance with Hamanishi et al. whereby a simplified formula for cross-sectional area is used utilizing coronal and sagittal diameters in accordance with the approximate geometric shape of the lumbar dural tube [13]. A cross-sectional area of the dural tube with 70–100 mm^2 ^was classified as moderate, and less than 70 mm^2 ^as severe spinal stenosis [6].

Descriptive statistics were computed for all parameters. All data was tested for deviation from the normal distribution within the groups using a skewness test and Box-and Whisker-Plots. Mean and standard deviation (± SD) were calculated for normally distributed variables, and median and interquartile ranges (IR) for non-normally distributed variables. Nominal and categorical values were expressed as absolute numbers and percentages. Group comparisons were performed using a two-sample t-test. Agreement between categorical objective and subjective assessment was expressed by Cohen's weighted Kappa. For bivariate statistics Kendall's Tau B coefficient was calculated in order to assess the correlation between walking distance, mean cross-sectional area of the overall dural tube, number of segments with a cross-sectional area of less than 70 mm^2^, age (> 65 years), functional back capacity score (FFbH-R), BMI, and depression status (statistical software SPSS 11.0. SPSS Inc. Chicago, Illinois, USA).

Multifactor analysis was carried out in a third step based on the categorical dependent variable -objective walking distance- using multiple ordinal regression analysis. In order to avoid over-fitting and to implement a procedure with the fewest possible parameters, all non-significant predictors were excluded after bivariate analysis.

All tests were two sided and a p value of 0.05 or less was considered statistically significant. Because of the explorative character of the study and incomplete theoretical background of current research, the authors did not make any adjustments to the significance level.

## Results

Sixty-three patients (37 women and 26 men) with a median age of 68 years (IR 13) and a mean BMI of 28 (SD 4.1) were included in our study. According to our inclusion criteria, patients suffered from leg pain dependent upon walking as indicated in table 1. The mean overall functional back capacity (FFbH-R) in the study population was 44% (SD 20.4, the age related norm value was 78).

**Table 1:** Descriptive statistics

**Dimension**	**Coding**	**Empirical Measurements**
		Arithmetic mean ± standard deviation^2 ^or frequency of entries

Number of patients		N = 63
Age	Patient Age	68.11 (13.18)
Gender	Male	26
	Female	37
Height	cm	167.92 ± 9.37
Weight	kg	78.49 ± 13.48
Body mass index (BMI)	kg/m^2^	27.81 ± 4.14
Functional capacity (FFbH-R)	Kohlmann (1996)	43.75 ± 20.41
Depression score (CES-D)	Radloff (1977)	13.63 ± 8.28
Area L1/2	mm^2^	113,65 ± 30.38
Area L2/3	mm^2^	93.79 ± 31.47
Area L3/4	mm^2^	73.00 ± 30.58
Area L4/5	mm^2^	65.16 ± 31.07
Area L5/S1	mm^2^	92.79 ± 32.70
Mean canal diameter of the dural sac	mm	87.68 ± 22.38
Number of segments with less than 100 mm^2^	--	1.84 ± 1.36
Number of segments with less than 70 mm^2^	--	1.65 ± 1.26
Lowest area of all segments	--	52.65 ± 452.20
**Subjective assessment**		
Location of pain^1^	Back pain	48
	Back pain and leg pain	52
Anticipation of leading pain location^1^	Mainly back pain	21
	Mainly leg pain	29
	Tiredness in the legs	20
Anticipation of pain quality^1^	Pulling pain	38
	Dull pain	12
	Burning pain	17
Behavior subsequent to pain aggravation^1^	To stand still	32
	Forward bending	15
	Sitting	28
Subjective estimation of walking distance (by category)	Up to 50 m	9
	< 50–100 m	7
	<100–200 m	13
	<200–500 m	15
	>500 m	19
**Gait analysis**		
Objectively measured walking distance (metric)	m	172.00 (422.00)
Walking time (seconds)	s	352.00 (500.00)
Maximum walking speed	m/s	0.60 ± 0.27
Objectively measured walking distance (by category)	Up to 50 m	9
	< 50–100 m	14
	<100–200 m	14
	<200–500 m	10
	>500 m	16

Due to the small numbers of patients percentages were not calculated.

1) Multiple answering

2) For the variables that are not normally distributed (age, absolute walking distance prior to treatment, seconds walking preoperatively) the median and in parentheses inter-quartile range are presented.

The depression status on the CES-D scale scored an average of 13.6 (SD 8.3, table 1), implying a depressive mood in 25 patients.

Patients' subjective walking distances were assessed prior to treadmill gait analysis as shown in table 1. We found a median walking distance of 172 m (IR 422) and a median walking duration of 352 seconds assessed by treadmill gait analysis. All patients stopped due to leg pain. The mean treadmill speed in the study population was 0.67 m/s. Twenty-three patients could walk less than 100 m, and 16 patients were able to walk more than 500 m. Twenty out of 63 patients correctly estimated their walking distance with a deviation of less than 50 m (table 2). However, 16 patients underestimated and 17 patients overestimated their absolute walking distance, implying that only every third patient (20 out of 63) was able to estimate his/her maximal walking distance correctly to within 50 m. The determined Kappa coefficient displayed a value of 0.121.

**Table 2:** Association between subjective and objective walking distance

Subjective estimation of walking distance (by category)	Objectively measured walking distance (by category)						Test of agreement (Cohen-kappa/significance)
	
	< 50 m	50–100 m	100–200 m	200–500 m	> 500 m	Total	
	
< 50 m	4	0	3	1	1	9	0.121/p = 0.024*
50–100 m	0	3	2	1	1	7	
100–200 m	2	7	1	2	1	13	
200–500 m	2	2	4	3	4	15	
> 500 m	1	2	4	3	9	19	
Total	9	14	14	10	16	63	

n = 63, significance limit: p < 0.05 (*)

The mean cross sectional area from the first lumbar through the first sacral level was measured according to Hamanishi et al., as described above, and is visualized in table 1[13]. The mean cross sectional area measured less than 100 mm^2 ^at an average of 1.84 out of five segments and less than 70 mm^2 ^at an average of 1.65 out of five segments.

### Correlation analysis

Results of correlation analysis determining the coherence of objectively measured walking distance and clinical and radiological parameters are visualized in table 3. The age and gender did not show any significant influence on the walking distance. A significant correlation could be shown between the BMI (tau b = -0.194, p = 0.025), the functional capacity (taub b = 0.225, p = 0.011), and the cross sectional area of the dural tube at L1/2 (tau b = -0.118, p = 0.032) with the objectively measured walking distance. Even though the depression status on the CES-D scale scored an average of 13.6 (SD 8.3) no significant correlations with the walking distance could be displayed.

**Table 3:** Influence of potential factors on the objectively measured walking distance

**Potential factors**	**Bivariate Analysis (with metric walking distance)**	**Multiple Analysis (Ordinal regession analysis with categorized walking distance)**
	Test parameter/significance	Test parameter/significance
Age	-0.024	--
Gender	425^1)^	--
Height	0.066	--
Weight	-0. 121	--
Body Mass index (BMI)	-0.194*	-0.079
Functional status (FFbH-R)	0.225*	+0.017
Depression Score (CES-D)	-0.053	--
Area L1/2	-0.188*	-0.017*
Area L2/3	-0.140	--
Area L3/4	-0.004	--
Area L4/5	-0.115	--
Area L5/S1	-0.058	--
Number of segments with less than 100 mm^2^	-0.106	--
Mean canal diameter of the dural sac mm^2^	-0.142	--
Number of segments with less than 70 mm^2^	0.029	--
More than two segments with less than 70 mm^2^	295^1)^	--
Lowest area of all segments	-0.113	--

n = 63, significance limit: p < 0.05 (*)

1) Test value from Mann-Whitney U test, otherwise Kendall's Tau B

The mean overall cross sectional area of all segments- the number of segments measuring either less than 100 mm^2 ^or less than 70 mm^2^- as well as the smallest dural area of each patient were not found to have an influence on walking distance (table 3).

The advantage of multiple regression analyses lies in their ability to maintain possible confounders at a constant value, which consequently helps to expose spurious correlations. The simultaneous influences of BMI, functional back capacity and area 1/2 upon maximal walking distance were assessed whereby a significant effect upon walking ability could only be found for area 1/2 (table 3). In order to reveal potential suppressor effects a second regression analysis including age and depression score was computed. This showed an equally significant effect of area 1/2 alone upon maximal walking distance with almost identical parameter values, consequently the results are not given here.

## Discussion

The prevalence of degenerative spine disease will increase with the aging of the population and symptomatic lumbar spinal stenosis will continue to be one of the most frequent indications for spinal surgery [1,10,14]. Despite the increasing socioeconomic impact of lumbar spinal stenosis an accurate diagnosis remains difficult to make. Studies evaluating the likelihood that surgery will be performed display wide geographic variations, highlighting the lack of specific and reliable diagnostic tools, and a clinical consensus regarding therapy [1,15].

The history of neurogenic claudication and assessment of radiological parameters continue to play a pivotal role in clinical practice, and serve as indicators for further therapeutic treatment such as surgery, even though the accuracy of these tools is controversial [7,14-17].

The aim of this clinical study was to determine the coherence of MR-imaging parameters and subjective clinical affliction, with the objectively measured walking distance in patients with symptomatic lumbar spinal stenosis, in order to examine if these parameters can serve as reliable tools in clinical decision making. Furthermore, the influence of clinical and sociodemographic parameters on the walking capacity was assessed in order to determine if symptomatic lumbar spinal stenosis displays a multi-factorial nature with respect to its causes, diagnosis, and treatment.

In our study the subjective assessment of functional performance in terms of the estimated walking distance differed from objective findings as shown in table 2. The determined Kappa coefficient indicates a significant agreement, but the value of 0.121 is relatively low. That confirms that the subjective assessment of maximal walking distance by the patients does not adequately reflect the reality. Thus, the subjective history of neurogenic claudication cannot serve as a reliable screening tool and leads to the conclusion that in addition to the evaluation of pain records, the walking distance should be verified by the clinical staff. Treadmill gait analysis can serve as a reliable established method if available [16,18,19].

Furthermore, our results could not reveal any significant correlations between the objectively measured walking distance using gait analysis, and the cross sectional area of the dural tube measured by MRI in patients with lumbar spinal stenosis.

The displayed correlation between the objectively measured walking distance and dural sac narrowing at L1/2 is not expected to be clinically relevant.

Our results are consistent with recent studies demonstrating that there is little relationship between central canal size and clinical symptoms among persons with a clinical diagnosis of LSS, and that MRI does not sufficiently differentiate between clinical spinal stenosis and controls [20,21]. In comparison to the study of Haig et al. and Geisser et al. our study population displayed more severe clinical symptoms. Taken together, this underlines that our results apply to a broad patient population independent of the clinical affliction.

The correlation between spinal stenosis and clinical symptoms has been the subject of continuing controversy. While some authors acknowledge a correlation only for certain groups of patients others have reported an influence of spinal canal dimensions in multilevel foraminal narrowing [1,3,17,22-24]. None of these studies demonstrated a clear association between the degree of narrowing and clinical symptoms nor could cutoff values be determined.

Furthermore, studies evaluating MR-imaging in asymptomatic patients demonstrate spinal narrowing in 21–28%, indicating a low coherence of MRI parameters and clinical symptoms in accordance to our study [25,26].

Some authors have attributed general fitness, age, muscle strength, and pain coping strategies to radiographic severity and non-operative outcome of patients with spinal stenosis [23]. Others correlated obesity and depressive situation with worse spine related symptoms [7,9,10].

Although several authors question the diagnostic accuracy of MRI findings because narrowing of the dural sac cannot easily be assessed due to specific imaging modalities (lack of spinal stress during scanning), it is the leading modality in the imaging of spinal disease. MRI is more likely to show bony as well as ligamentous structures without the risks and discomfort of myelograms [11,27].

Correlation analysis of clinical parameters, the depression status and the subjective functional back capacity with the absolute walking distance are shown in table 3. In bivariate analysis, a reduced functional capacity (FFbH-R) correlated with the absolute walking distance on the treadmill. However, as only one out of the 12 items of the FFbH-R refers to running, further parameters of behavioral dysfunction in respect to avoiding pain and walking should be examined. Moreover, our data displayed a significant negative correlation between walking distance and BMI. Takahashi et al. assumed that obesity provoked lordosis and led to a narrowing of the spinal canal with subsequent aggravation and functional loss [28]. It should be noted that people who are overweight (BMI > 25) suffer more frequently from back pain than people with normal weight [7].

Forty percent of our patients suffered from depression, even though no correlation between depression and walking distance could be demonstrated in this study. Depression is reported to play a significant role in patients with unspecific chronic back pain [37]. Chronic back pain is known to display a multi-factorial nature often accompanied by emotional distress [29,30]. The high number of patients with depression may lead to the suggestion that depression may play a more active part in LSS than previously thought.

Fear avoidance behavior can be seen as the primary dysfunction in daily activities of patients suffering from unspecific low back pain [12,31,32]. The concurrence of FFbH-R, depression, and elevated BMI in our study suggests that patients may have noticed a restriction in walking ability after some 100 meters and attempt to avert pain through avoidance behavior by reduction of walking distances. Continuing avoidance of walking leads to further reduction in functional parameters, and body mass increases which in turn leads to depressive cognition and further avoidance behavior. Another explanation would be the lack of reliable diagnostic tools to clearly identify patients with lumbar spinal stenosis. This leads to the conclusion that the increasing diagnosis of LSS also contains patients who indeed display a spinal canal narrowing in radiological imaging, but suffer primarily from unspecific chronic back pain. This might be one reason why patients' satisfaction in terms of walking improvement after surgery including decompression of the spinal canal is limited, and therapy failures of 20–40% after decompressive surgery are reported in long-term outcome evaluations [4,16,33,34].

However, in multivariate analysis, FFbH-R, depression, BMI, and central spinal stenosis were not statistically significant independent predictors for walking distance.

Our results point out that the patients' prognosis might depend upon additional factors, such as an increased BMI together with reduced functional capacity and ability to overcome fear avoidance. [4,15,35].

This is in accordance with studies showing that functional performance can simply be improved with non-surgical treatment options such as general fitness programs or pain coping strategies, but, of course, the pathomorphology remains unchanged [1,6,9,13,23,24]. A recent longitudinal study demonstrated improved function and a decrease of pain in patients with LSS, without receiving any surgical intervention [36]. This demonstrates that anatomy does not predict functional ability and emphasizes the idea that interventions to address pain and function may be more successful than those that manage anatomy. The influence of psychosocial issues should be evaluated before surgery and taken into consideration in the therapy concept. Therapy modalities, which strengthen a passive patient's attitude, should be omitted. In addition to weight reduction, an age-related cognitive behavioral therapy such as a multidisciplinary approach, should be chosen in order to compensate avoidance behavior as expressed in the low rated scores in functional back capacity. This would be consistent with our experience with therapy for chronic pain and disability in the aged population [38].

Some comments can be made on the population and the methods used in this study. The assessment of patients with symptomatic spinal stenosis remains difficult since the population is heterogeneous and patients often present with a high number of comorbidities. As described above we tried to address this issue by performing diagnostics to create a more homogeneous population, which is a major strength of this study.

The lack of objective research methodologies is a major deficit of the studies evaluating patients with spinal stenosis, and potentially, issues of mental overlay could render the results bias. We tried to balance this issue by using reliable tests and independent staff to objectively assess gait analysis and MRI data sets. However, before making any generalizations, it must be taken into account that a relatively high number of our study population suffered from depression. The influence of psychosocial issues on pain and disability in patients with LSS is likely huge, but relatively unstudied. Further research is needed to address this issue. A limitation of the study is the small number of patients (n = 63) and the consequent risk of a Type II error. With the given 63 complete data sets which were included in the analyses and 3 independent variables, small effects could probably not have been detected. It was attempted to minimize the influence of further potential effectors with inclusion and exclusion criteria.

A post hoc performed power analysis showed (1-beta)-values of less than 0.80. The restriction to few clinically relevant variables such as factors determining walking distance was accepted in order to counter this.

## Conclusion

According to the results of this study, the subjective history of limited walking distance has to be verified by the medical staff prior to decision making in order to improve the identification of patients with symptomatic lumbar spinal stenosis and distinguish them from those with unspecific chronic back pain.

A correlation between the objectively measured walking distance and the dural tube narrowing of all segments could not be confirmed by our data, implying that MRI findings seem to have less clinical relevance on the walking distance in patients with symptomatic lumbar spinal stenosis than previously assumed. In determining the disease pattern of lumbar spinal stenosis, functional back capacity and BMI might play a more active role than previously thought, indicating that lumbar spinal stenosis may display a multi-factorial nature with respect to its causes, diagnosis, and treatment.

## Competing interests

The authors declare that they have no competing interests.

## Authors' contributions

FZ and BM have made substantial contributions to conception and writing and did the data analysis and drafted the manuscript. MS have helped in designing the study and critically revised the manuscript. RA helped performing the gait analysis. All authors read and approved the final manuscript.

## Pre-publication history

The pre-publication history for this paper can be accessed here:


